# The Effect of Thermoplastic Elastomer and Fly Ash on the Properties of Polypropylene Composites with Long Glass Fibers

**DOI:** 10.3390/polym16091238

**Published:** 2024-04-29

**Authors:** George Mihail Teodorescu, Zina Vuluga, Rodica Mariana Ion, Toma Fistoș, Andreea Ioniță, Sofia Slămnoiu-Teodorescu, Jenica Paceagiu, Cristian Andi Nicolae, Augusta Raluca Gabor, Marius Ghiurea

**Affiliations:** 1 National Institute for Research & Development in Chemistry and Petrochemistry—ICECHIM, Research Group 12—Polymeric Composites and Nanocomposites, 202 Splaiul Independentei, 060021 Bucharest, Romania; rodica.ion@valahia.ro (R.M.I.); toma.fistos@icechim.ro (T.F.); andreea.afilipoaei@icechim.ro (A.I.); cristian.nicolae@icechim.ro (C.A.N.); raluca.gabor@icechim.ro (A.R.G.); marius.ghiurea@icechim.ro (M.G.); 2 Doctoral School of Materials Engineering Department, “Valahia” University of Targoviste, 35 Lt. Stancu Ion, 130105 Targoviste, Romania; 3 Materials Engineering and Mechanics Department, Valahia University of Targoviste, 13 Aleea Sinaia, 130004 Targoviste, Romania; sofiateodorescu@yahoo.com; 4 CEPROCIM S.A., 6 Preciziei, 062203 Bucharest, Romania; jenica.paceagiu@ceprocim.ro

**Keywords:** polypropylene, long glass fiber, fly ash, thermoplastic elastomer, mechanical properties

## Abstract

A cost-effective solution to the problems that the automotive industry is facing nowadays regarding regulations on emissions and fuel efficiency is to achieve weight reduction of automobile parts. Glass fiber-reinforced polymers are regularly used to manufacture various components, and some parts may also contain thermoplastic elastomers for toughness improvement. This work aimed to investigate the effect of styrene-(ethylene-co-butylene)-styrene triblock copolymer (E) and treated fly ash (C) on the morphological, thermal, and mechanical properties of long glass fiber (G)-reinforced polypropylene (PP). Results showed that the composites obtained through melt processing methods presented similar thermal stability and improved (nano)mechanical properties compared to 25–30 wt.% G-reinforced PP composites (PP-25G/PP-30G). Specifically, the impact strength and surface hardness were greatly improved. The addition of 20 wt.% E led to a 25–39% increase in impact strength and surface elasticity, while the addition of 6.5 wt.% C led to a 16% increase in surface hardness. The composite based on 25 wt.% G, 6.5 wt.% C, and 20 wt.% E presented the best-balanced properties (8–17% increase in impact strength, 38–41% increase in axial strain, and 35% increase in surface hardness) compared with PP-30G/PP-25G. Structural and morphological analysis confirmed the presence of a strong interaction between the components that make the composites. Based on these results, the PP–G–E–C composites could be presented as a viable material for automotive applications.

## 1. Introduction

Nowadays, automobile manufacturers face great challenges because of strict regulations governing fuel efficiency and CO_2_ emissions. Because there are currently few technologies available to increase fuel efficiency, alternative solutions, such as reducing the weight of automotive components, are needed. A study conducted by D’Errico and Ranza [1] estimated that every 10% reduction in automobile weight would improve fuel economy by 7%. Weight reduction of automobile parts is the most cost-effective method to reduce fuel consumption and can be achieved through the optimization of structure, improving the functionality of components, and employing lightweight materials. A commonly used polymer in both interior and exterior applications for automobiles in the form of components is polypropylene due to its high chemical resistance, imperviousness to water and high UV resistance. Polypropylene is frequently used to obtain composites and nanocomposites because it can be processed using industrial technologies, including injection molding and extrusion [2]. An approach that may lead to obtaining lightweight materials is to introduce fillers/nanofillers as reinforcing agents while reducing the glass fiber content in composites. Small loadings (under 5% by weight) of fillers are becoming more common in the development of lightweight materials, as noted by Garcés et al. [3] where nanofillers are expected to improve properties such as modulus, thermal stability, surface hardness etc., and eventually replace metal components, leading to durable and lightweight vehicles in the automotive industry. In recent years, there has been a rise in curiosity regarding the application of fly ash, a byproduct of the burning of coal. This ash consists of silico-aluminous components and is formed at temperatures of 920–1200 °C. It is essentially a residue made up of solid particles (precipitator ash) and small, hollow spheres (cenospheres) that are captured by collection ponds. This material needs a lot of land, water, and energy to be managed and disposed of. Fly ash comes in the form of fine particles that, if handled improperly, can lead to their spreading in the air, thus posing a serious threat to public health. Several studies have been performed to introduce this waste material as a low-cost filler by itself or in various combinations with organic or inorganic materials [4,5,6,7]. In addition to these studies, fly ash was discovered to act as a reinforcing agent in a study by Satheesh et al. [8], where good interaction between the ash and the matrix was achieved, while a study by Nath et al. [9] and Gummadi et al. [10] pointed out that the properties of PP composites with fly ash content are dependent on the particle size of said ash. In a study performed by Kutchko and Kim [11], it is pointed out that smaller-particle-size fly ash leads to better mechanical properties as long as the particles are well dispersed in the polymer matrix. In a previous investigation by Teodorescu et al. [12], the characteristics of composites obtained from silico-aluminous industrial waste, similar in composition to fly ash, and recycled polypropylene were studied. Results showed improved thermal and mechanical properties as well as good adhesion between the small particles of ash and the polymer matrix due to the presence of compatibilizing and dispersion agents.

Composite dispersion and compatibility agents are frequently needed to produce a homogeneous mixture, especially when working with materials that have weak polar reactive groups. Polypropylene would need a dispersion as well as a coupling agent because of its hydrophobic nature and limited compatibility with aluminosilicates such as ash powder. To enhance the interfacial interaction between the polymers and the fillers, commercial macromolecular compatibilizers (polyolefins treated with maleic anhydride) are frequently utilized as shown by Chrissopoulou and Anastasiadis [13] and Joseph et al. [14]. At the same time, agents like poly(propylene glycol adipate) can be employed to improve the dispersion of fillers in the polymer matrix, as shown in studies by Hao et al. [15] and Zhao et al. [16], where this dispersion agent was shown to act as a plasticizer and improve the compatibility between the composite components and the general mechanical properties.

Glass fiber is used as a reinforcing agent in polymer composites due to its capacity to provide increased stiffness, dimensional stability, and modulus to these materials. Glass fiber-reinforced plastics are already used as an alternative material for lightweight automobile parts by replacing metallic materials. There are studies on the preparation of polymeric composites reinforced with glass fiber where the reinforcing agent was added to the polypropylene matrix with the purpose of improving the mechanical properties of the final composite and to reduce its cost [17]. However, a major disadvantage of glass fiber is the high loading levels that must be used to achieve the desired properties and the difficulty of recycling it. In this sense, studies by Wu et al. [18] and Joshi [19] were performed seeking to reduce or replace the short glass fiber content in automotive parts for reduced energy consumption and improved environmental impact. Emerging trends in lightweight materials development are to continue to develop automotive composites reinforced with other performing but cheaper reinforcement agents such as fly ash.

To obtain parts with improved specific properties such as high elasticity, materials such as thermoplastic elastomers are employed. Thermoplastic elastomers belong to a class of materials that exhibit properties typical of rubbery materials, but have the combined physical properties of thermoplastics and elastomers and can be processed like thermoplastics. Styrene–ethylene–butylene–styrene (SEBS) thermoplastic elastomer is a regularly used elastomer in a wide variety of general-purpose rubber items, as well as in handlebar grips, toothbrushes, sports mouth guards etc. SEBS can improve the deformability and elastic mechanical properties of composites due to its long polymeric chains, which are joined into a network structure and have a high degree of mobility and flexibility.

As of now, there are very few studies that have investigated the interaction between fly ash and other reinforcing agents such as long glass fibers in a polymer matrix. In a previous study by Vuluga et al. [20], the effect of SEBS on the mechanical and thermal properties of polypropylene was investigated. The results showed an increase in toughness, expressed by an increase in energy at break and impact strength values, as well as an increase in elongation. Another study [21] investigated the effect of SEBS–halloysite masterbatch on the properties of hybrid polypropylene–glass fiber composites under optimal processing conditions. Improved hardness, modulus, tensile strength and impact strength were obtained for the polypropylene–chopped glass fiber composites containing masterbatches compared to pristine PP. A study by Panaitescu et al. [22] investigated the effect of SEBS on polypropylene composites filled with nanosilica particles, where the results showed a simultaneous increase in all tensile and impact characteristics in the case of the composite blend compared to neat PP. A similar investigation done by Sanporean et al. [23] researched the properties of polypropylene–organoclay–SEBS nanocomposites and showed that the optimal ratio between SEBS and organosilicate in the polypropylene matrix can lead to improved toughness or stiffness for the nanocomposites and even a good balance between the two. These articles create a basis for the present study, as interactions between PP and SEBS or between PP and GF/nanofillers have already been studied. To our knowledge and compared to our previous articles, there have been no studies to investigate the interaction between fly ash powder, long glass fiber, and polymers containing a thermoplastic elastomer as an impact modifier. Therefore, the present study can lead to several potential research directions to develop various composites with specific applications in such areas as the automotive industry. Compared to our previous work, the novelty of this article consists in the use of polypropylene reinforced with long glass fibers, a specific type of ash and a thermoplastic elastomer and studying the interaction between the components and the effect of each on the properties of the final composite.

The main purpose of our work was to analyze the characteristics of long glass fiber-reinforced polypropylene, elastomer and fly ash composites to indicate a cheaper but resourceful alternative for partial replacement of glass fiber and obtain new composites with a good balance of stiffness–toughness properties. Replacement of a percentage of glass fiber with fly ash and addition of thermoplastic elastomer will be monitored to determine if the ratio between components in the final compositions can provide similar or improved mechanical properties to commercial glass fiber-reinforced polypropylene composites commonly used in the automotive industry. This reduction in the glass fiber content must be accomplished without compromising the necessary properties of these composite materials for automotive applications.

## 2. Materials and Methods

### 2.1. Materials

A commercial high-flow copolymer polypropylene (PPBJ100HP, Borealis, AG, Vienna, Austria) with density ρ = 0.904 g/cm^3^ and melt flow index (230 °C/2.16 kg) = 90 g/10 min was used as the polymer matrix. Nepol GB601HP, a special long glass fiber-reinforced polypropylene concentrate grade (Borealis AG, Vienna, Austria), was diluted with PPBJ100HP until 25% and 30% glass fiber (G) concentration was achieved. An aluminosilicate fly ash powder sourced from the Govora thermal power plant (CET Govora S.A., Râmnicu Vâlcea, Romania), was used as reinforcing filler along with G. A linear thermoplastic elastomer styrene–ethylene–butylene–styrene triblock copolymer (KRATON 1652G, Kraton Polymers, Houston, TX, USA), marked with E, with 29% styrene content and melt flow index = 5.0 g/10 min (230 °C/5 kg) was used to enhance the elasticity and impact strength of the researched composites. Additionally, poly(propylene glycol adipate) (Solventul S.A., Timisoara, Romania) with viscosity at 20 °C: 11000–16000 cP and density at 20 °C = 1.150–1.200 g/cm^3^ was used as a dispersion agent and polypropylene–graft–maleic anhydride (Polybond 3200, Crompton, Middlebury, VT, USA), marked with MA, characterized by a melting point of 157 °C and density of 0.91 g/cm^3^ was used as compatibilizing agent.

### 2.2. Preparation of Composites

The preparation of fly ash as a reinforcing filler in polymer composites and its treatment with a dispersion agent was discussed in detail in a previous article by Teodorescu et al. [12]. In that study, the ash powder was dried, milled and sieved to achieve particle sizes <90 µm and moisture content <0.1%. The oxidic chemical composition determination for this fly ash revealed two major components: SiO_2_ (56.24%) and Al_2_O_3_ (22.44%). The surface of fly ash was treated with poly(propylene glycol adipate) and the modified ash powder, marked with C, was characterized by SEM, EDX, X-ray diffraction and FTIR analysis [12]. The treated ash powder and the glass fiber, compatibilizing agent, and thermoplastic elastomer were dispersed and mixed within the polypropylene matrix (marked with PP) using a Leistritz LSM 30.34 co-rotating twin screw extruder (Leistritz Extrusionstechnik GmbH, Nürnberg, Bayern, Germany) at a temperature of 180-205 °C (ranging from hopper to die), 220 rpm screw rotation speed and 450 rpm feeding rate. The resulting filaments were cooled in a water bath and then granules were obtained using a Leistritz Granulator system (Leistritz Extrusionstechnik GmbH, Nürnberg, Bayern, Germany). Standard injected specimens for impact and tensile tests were obtained by injection molding using an Engel 40/22 (Engel, Schwertberg, Austria) with an injection speed of 2 mm/min, a temperature profile of 220 to 240 °C, mold temperature of 50 °C and pressure of 600 kg/cm^2^. The final composite formulations are presented in Table 1.

### 2.3. Characterization

#### 2.3.1. X-ray Diffraction

A Rigaku Smartlab (Rigaku Corporation, Tokyo, Japan) diffractometer was employed to take X-ray diffraction data, utilizing CuKα1 (λ = 1.5406 Å) radiation. The generator radiation’s acceleration voltage was set at 45 kV, while the emission current was 200 mA in this test. Room temperature diffractograms were acquired using parallel beam geometry, scanning 2θ = 5° to 60° at increments of 0.02° in a continuous manner at a scanning pace of 4°/min. The Bragg equation was used to determine the interplanar distance (d) of PP in composites:

(1) nλ=2dsin⁡θ

Here, n is the reflection order, θ is the diffraction angle, λ is the wavelength of X-rays, and d indicates the distance between the crystal lattice planes, which cause the diffraction. The margin of error was ±0.05%.

Rigaku PDXL 2 data analysis software was used to obtain the crystallite sizes, height, intensity values and FWHM (full width at half-maximum intensity of the diffraction peak). Crystalline phases were identified according to the Powder Diffraction File^TM^ (PDF) of the International Centre for Diffraction Data (ICDD). Curve fitting applied to diffraction profiles was used to extract individual diffraction maxima. Using a peak deconvolution program (PDXL: integrated X-ray powder diffraction software), the amorphous phase was assumed to be represented by a broad peak at roughly 2θ of 18°. By reporting the area of the maximum diffraction of the crystalline phases to the total area (crystalline and amorphous phases) resulting from the elimination of the reference line, the crystallinity index (CI) was calculated with a margin of error of ±2%. The orientation index (OI) was calculated with a margin of error of ±5% using the relationship:

OI = I_040_/I_110_ − 0.55 (2)

where I_040_ and I_110_ represent the height of the (040) diffraction peak and the height of the (110) diffraction peak, respectively, and 0.55 is the ratio of I_040_ and I_110_ determined for a perfect non-oriented PP. The mean size of the crystalline domains was determined based on the Scherrer equation:

(3) $$B(2\theta )=\frac{K\lambda }{Lcos\theta }$$

where B(2θ) is the size of the ordered crystalline domains for the Bragg angle in question, K—a dimensionless shape factor (0.9), L—full width at half maximum (FWHM), λ—X-ray wavelength (1.54059Å), and θ—Bragg angle.

#### 2.3.2. Fourier-Transform Infrared Spectroscopy (FTIR)

The FTIR spectra were drawn utilizing a Vertex 80 from Bruker (Bruker Optics GmbH & Co. KG, Ettlingen, Germany), provided with a diamond crystal module with total attenuated reflection (ATR), 0.2 cm^−1^ spectral resolution and accuracy of 0.1% T. These were registered in the transmission mode (32 scans) in the range 4000–400 cm^−1^ without any preparation, the data were collected using Opus software, and the absorbance mode was calculated and represented vs. wavenumber.

#### 2.3.3. Thermal Characterization

Thermogravimetric analysis (TGA) was conducted using a TA-Q5000IR (TA Instruments, New Castle, DE, USA). As purge gas, nitrogen at a rate of 40 mL/min was used. Samples of 7–8 mg were heated from 25 °C to 700 °C at a rate of 10 °C/min.

To measure the melting and crystallization properties of the samples, a differential scanning calorimetry (DSC) equipment (Q2000, TA Instruments, New Castle, DE, USA) was used. A heat–cool–heat (HCH) method was employed in a single run, beginning with a 3 min equilibration at −40 °C. Then, the sample was heated to 200 °C, held there for 2 min, cooled to −40 °C, held there for another 2 min, then heated back up to 200 °C at a rate of 10 °C/min and under 5.0-grade helium at a flow rate of 25 mL/min.

#### 2.3.4. Dynamic Mechanical Analysis (DMA)

The storage modulus (E′), loss modulus (E″), and loss factor (tan δ) of the samples were determined using a DMA Q800 (TA Instruments, New Castle, DE, USA) in relation to temperature. A temperature range of −70 to 120 °C was scanned on samples with dimensions of 35 × 10 × 4 mm (length, width, and thickness) using 20 µm amplitude, 1 Hz frequency, and a 3 °C/min heating rate.

#### 2.3.5. Mechanical Analysis

The tensile characteristics of the samples were evaluated using the Instron 3382 universal testing machine (Instron Corporation, Norwood, MA, USA) in accordance with ISO 527 [24]. For each test, seven specimens were used, and the tensile strength was measured at 50 mm/min and the modulus of elasticity at 2 mm/min. Using a Zwick HIT5.5 Pendulum Impact Tester (Zwick Roell AG, Ulm, Germany), seven specimens per test, and the Charpy notched impact method in accordance with ISO 179-1/1 e A [25], the impact strength of the samples was calculated.

#### 2.3.6. Nanomechanical Analysis

Using a three-sided pyramidal Berkovich tip, testing for nanoindentation and nanoscratching was carried out using a TI Premier system (Hysitron Inc., Minneapolis, MN, USA) (radius of curvature of 150 nm and total angles of 142.35 deg). Samples were mounted on metal plates and cleaned with 96% ethanol to remove all surface impurities such as dust and fats. Twenty indentations at a set force were performed per sample to acquire a wide range and accurate data. Load–displacement curves were recorded at a force of 10,000 µN while applying a trapezoidal load function—5 s loading, 2 s hold, 5 s unloading—and by using the Oliver-Pharr method, the values of hardness (H) and reduced modulus (Er) were obtained.

#### 2.3.7. Scanning Electron Microscopy (SEM)

The SEM analysis of the polypropylene composites’ morphological characteristics was conducted using a Hitachi TM4000 Plus microscope (Hitachi, Tokyo, Japan) at a 15 kV accelerating voltage. Before the SEM analysis, the active zone of the tensile test specimens was separated and submerged in liquid nitrogen. After 1 min, the samples were taken out and a pair of pliers was used to fracture the specimen-selected zone into approximately equal-sized fragments. The resulting fragments were coated with a 5 nm layer of gold using a Q150R Plus (Quorum Technologies, SXE, Lewes, UK).

## 3. Results and Discussion

### 3.1. X-ray Diffraction Analysis

**Figure 1:**
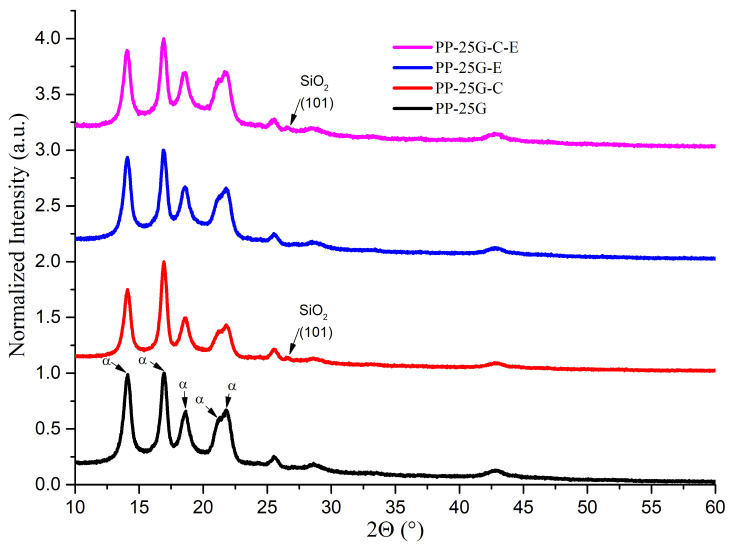
XRD patterns for PP composites in comparison with PP-25G.

XRD patterns for αPP in composites are shown in Figure 1, identified according to a study by Foresta et al. [26]. The α phase was recognized in PP-25G at 2θ = 14.09°, 16.94°, 18.59°, 21.21° and 21.88°, which are associated with the (110), (040), (130), (111) and (13-1) crystallographic planes. A study by Zhang et al. [27] showed similar diffraction peaks and crystalline planes for the α-form crystal of PP and indicated that PP–glass fiber composites processed by injection molding have proper orientation of polymeric chains. Kantz et al. [28] and Hnatkova and Dvorak [29] demonstrated that the injected PP samples exhibit a skin–core morphological structure that depends on both the intrinsic properties of the PP and the processing conditions. They found the existence of three crystalline layers on the thickness of the injected sample (a layer strongly oriented in the direction of the melt flow (non-spherulitic skin)), with thicknesses up to about 400 nm on the surface, a microspherulitic intermediate layer with a large number of nucleated spherulites at some depth, and a layer (core) composed of spherulites with large dimensions and low orientation in the middle. In the case of our composites, we expected that the morphological structure of PP would be influenced by the different effect of each component on the nucleation, crystallization rate, orientation and size of the spherulites of the αPP. With the addition of treated ash powder, the presence of another peak can be seen in PP-25G-C and PP-25G-C-E composites at 2θ = 26.55° and 26.53°, respectively, which correlate with the (101) SiO_2_ crystallographic plane (PDF card No. 01-090-3640). It is also observed that by incorporating the ash, the height of the (040) diffraction peak increases a lot compared to the (110) diffraction peak, which remains almost unchanged. This behavior demonstrates that the ash affects the orientation of the αPP crystallites. A similar effect was reported by Denac et al. [30] when incorporating talc in iPP. They found that talc crystals disrupt the spherulitic morphology of PP and orient in a plane parallel to the surface. The CI and the OI of αPP, calculated from X-ray diffraction, showed highest values for the composite PP-25G-C, evidence of the interaction between PP, MA, G and C and low chain mobility. In this case, the adhesion at the polymer matrix–filler interface is stronger due to the additional interactions between the dispersion agent and each of the composite components. Thus, the surface-treated fly-ash, C, can interact with PP through propylene groups and with PP-MA through -OH and ester groups from poly(propylene glycol adipate). After the addition of elastomer, both the crystallinity index and orientation index showed diminished evidence of the interaction between PP and E. This could be explained by the recrystallization of a part of the αPP crystals with the change in orientation direction. The high nucleation capacity of SEBS at the interface with iPP and the opposite effect it has on the orientation of crystallites with respect to minerals are known [30]. The addition of SEBS can result in a complex morphology consisting of both transcrystalline layers on the surface and radial spherulites inside the sample. It should be noted, however, that the OI is higher in the case of composites that, in addition to glass fiber, also contain other additives compared to composites with only 25% glass fiber. From Table 2, we see that the peaks that are characteristic for the α crystalline phase of PP present dissimilar intensities for PP-25G-C (the highest values for the height of the (110) and (040) diffraction peaks, so increased concentration of αPP) and PP-25G-C-E (the lowest values for the height of the (110) and (040) diffraction peaks) compared to PP-25G. These results highlight the existence of a strong interaction between PP, G, C and E especially in the PP-25G-C-E composite, partially due to the presence of dispersion and compatibilizing agents. In this composite, it is possible that the ash particles are oriented on the surface in a parallel plane and the elastomer particles are randomly distributed [30]. The analysis of the diffraction peaks showed no noticeable alterations in the crystalline structure of PP-25G composites. However, some changes were observed in the interplanar distances and the width at half-maximum intensity of the crystalline peaks. There seems to be a slight increase in interplanar distances (the concentration of defects in the crystals increases) beyond what can be attributed to experimental errors, particularly for PP-25G-E and PP-25G-C-E (Table 2). This implies a possible penetration of block copolymer particles into the spherulitic structure of PP, as a similar result was obtained in a study by Vuluga et al. [20]. The expected decrease in crystallinity for PP-25G-E and PP-25G-C-E composites in comparison to PP-25G can be attributed to the impact of the elastomeric phase interfering with the crystallization process of PP. This interference is particularly noted in cases where ethylene–butylene blocks are interpenetrating the PP structure. The XRD crystallinity index was 66% for PP-25G, 70% for PP-25G-C, 63% for PP-25G-E and 58% for PP-25G-C-E. It can be noticed that the CI decreases with the increase in composite complexity. The sample PP-25G-C has the highest values for CI and OI as well as larger crystals, and maybe, according to Nurul Huda et al. [31] and Parenteau [32], increased imperfection. We expect this behavior to be reflected in decreased toughness (impact strength and elongation at break). On the contrary, in the case of the PP-25G-C-E composite, which showed the lowest value for CI, we expect improved toughness (increased impact strength and elongation at break) as well as an improvement in surface hardness.

The FWHM data in Table 2 show small variations across all composites with an exception at the (111) crystallographic plane. PP-25G and PP-25G-E have higher values for FWHM, hence smaller crystals [27], compared to PP-25G-C and PP-25G-C-E. Studies by Vashista and Paul revealed an increase in the FWHM of the X-ray diffraction peaks due to the presence of residual surface stress [33].

### 3.2. FTIR Analysis

**Figure 2:**
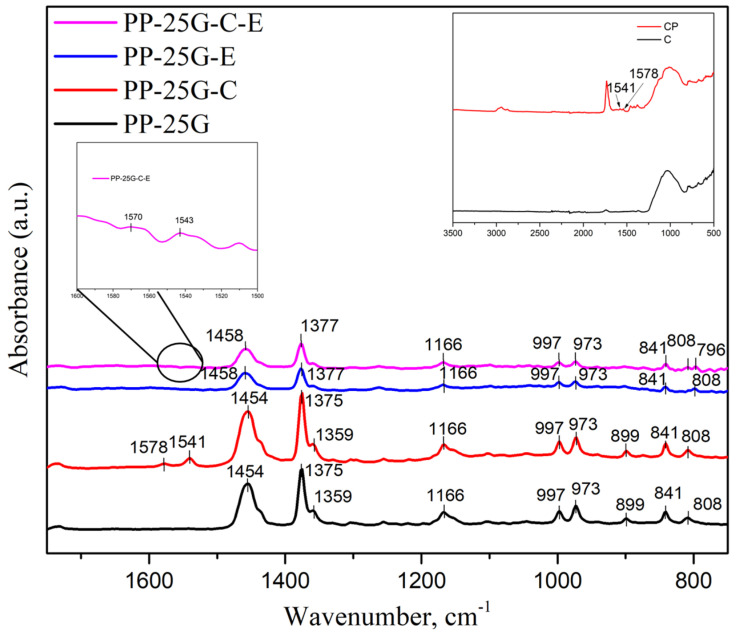
FTIR spectra of PP-25G, PP-25G-C, PP-25G-E and PP-25G-C-E composites.

The FTIR spectra for the PP composites with glass fiber, ash and elastomer are shown in Figure 2. The first piece of information that Figure 2 provides is that the peaks for PP-25G-E and PP-25G-C-E have a lower intensity compared to PP-25G-C and PP-25G. This is to be expected as PP-25G-E and PP-25G-C-E have lower PP content, as shown in Table 1. The peaks indicated in Figure 2, ranging from 973 cm^−1^ to 997 cm^−1^, are all related to the -CH_3_ asymmetric rocking vibration and C-C asymmetric stretching vibration of PP. Similarly, the peaks highlighted at 841 cm^−1^ are linked to the -CH_2_ rocking vibration of PP. The peak found at 997 cm^−1^ is associated with the crystalline phase of PP (α and β forms), while the 973 cm^−1^ peak is connected to both the crystalline and amorphous phases of PP [34]. The degree of crystallinity calculated from the ratio of the maximum intensities of the two peaks [29] varies in the order PP-25-E > PP-25G-C-E > PP-25G-C > PP-25G. The peaks at 1166 cm^−1^ can be associated with the rocking vibration of -CH_3_ while the peaks ranging from 1375 cm^−1^ to 1458 cm^−1^ are related to the symmetrical bending vibration of -CH_3_ [35,36]. In the case of composites containing elastomer, PP-25G-E and PP-25G-C-E, a slight shift of these peaks to the left is observed, proof of the interaction between PP and the ethylene–propylene blocks in the elastomer. New FTIR bands at 1578 and 1541 cm^−1^ were observed in Figure 2 for PP-25G-C and at 1570 and 1543 cm^−1^ for PP-25G-C-E, being specific to the addition of the treated ash additive, more specifically from the dispersion agent used for the fly ash surface treatment [12], and can be associated with the -COO^−^ antisymmetric stretching vibration group [37,38,39].

### 3.3. Thermogravimetric Analysis

**Figure 3:**
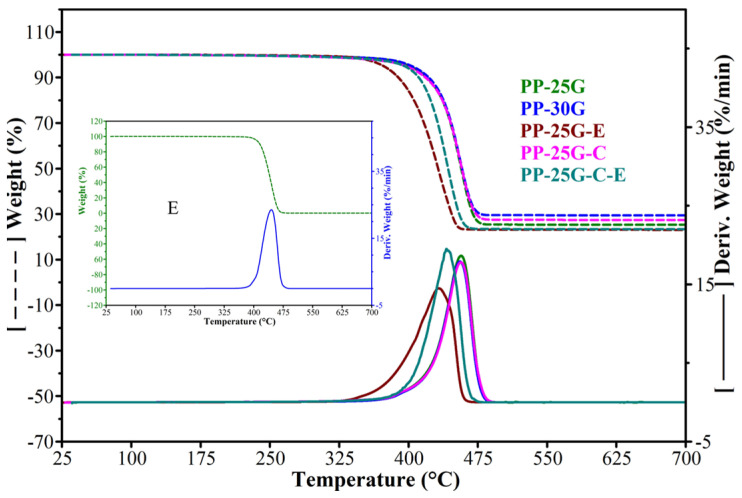
TGA and DTGA curves of E, PP-25G, PP-30G, PP-25G-E, PP-25G-C and PP-25G-C-E composites.

According to the TGA data (Figure 3 and Table 3) a single weight-loss step was observed for all samples between 300 and 500 °C. The thermal stability of the 25% and 30% glass fiber composites is similar to that of the composite with treated ash powder and glass fiber where the temperature at the maximal rate of decomposition only varies by a maximum of 1 °C. The lowest thermal stability is associated with PP-25G-E, with a difference of almost 24 °C compared to PP-25G and PP-30G. A study by Zhu et al. [40] noted that the addition of glass fiber in neat polypropylene can lead to an increase in thermal stability of polypropylene composites due to possible changes in the PP crystalline structure and the improved transfer of heat characteristics of glass fiber. It can be seen from Table 3 that E starts to decompose and decomposes at the maximum rate at temperatures 12 °C lower than PP-25G. Added to PP-25G, E interacts through the ethylene–butylene blocks with PP and affects its crystallization process, having a strong nucleation effect. This behavior would be due to an under-cooling effect, which is reflected in the decrease in Tonset. The addition of treated ash powder in the final composite (PP-25G-C-E) leads to an increase in thermal stability compared to PP-25G-E and a smaller difference (16 °C) between this composite and the glass fiber ones (PP-25G and PP-30G). The decomposition temperature of C far exceeds that of PP-25G, and as pointed out from EDX analysis and from previous determined chemical oxidic composition [12], C consists mostly of Si. It is possible that the silica particles strongly restricted the thermal decomposition of PP. Similar to a study by Zoukrami et al. [41], good dispersion of silica particles between PP chains caused by the addition of compatibilizing and dispersion agents may also lead to an enhancement of thermal stability. In our case, there is a strong interaction between the surface-treated ash, the compatibilizing agent and the PP matrix through propylene, -OH and ester groups from poly(propylene glycol adipate). The XRD results presented in Section 3.1 proved an increase in the crystallinity and orientation indices as well as the crystallite size, which is reflected in an increase in thermal stability. A previous study by Teodorescu et al. [12] also showed similar improvements in thermal stability for recycled PP with the introduction of ash powder. This behavior is also confirmed by the data in Table 2, where the temperature at which the samples lose 5% of their weight has small variations between PP-25/30G, PP-25G-C and PP-25G-C-E (5–8 °C) and large variations between PP-25/30G and PP-25G-E (29–32 °C).

### 3.4. Differential Scanning Calorimetry Analysis

**Figure 4:**
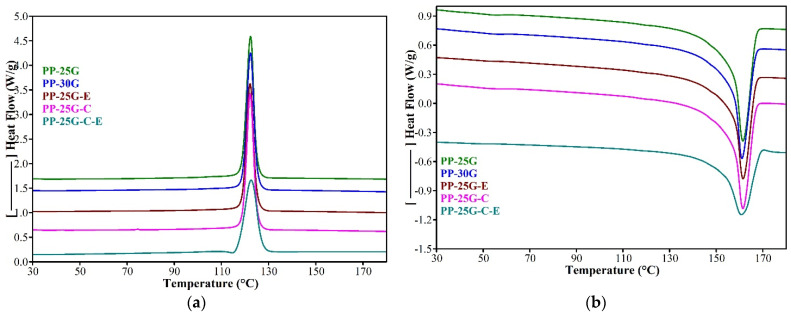
DSC thermograms of PP-25G, PP-30G, PP-25G-E, PP-25G-C and PP-25G-C-E composites: (**a**) cooling cycle; (**b**) second heating.

Figure 4 illustrates the DSC thermograms for PP composites reinforced with glass fiber, elastomer, and treated ash powder. Notably, the primary findings that are specific to the α crystalline phase of PP, Tc (maximum of the crystallization exotherm), Tm (maximum of the melting endotherm) and melting/crystallization enthalpy (ΔHm/ΔHc), are laid out in Table 4. DSC analysis showed that the Tm of the composites has very small variations across the composites by 0.4–0.7 °C, while Tc does not change. The lowest value for Tm was observed for the PP-25G-C-E composite. This behavior could be explained by the simultaneous influence of fly ash, SEBS, PP-MA, and glass fiber on the chain mobility of PP. From the DSC and XRD results it can be seen that Tm is affected by the crystallite size rather than percentage crystallinity. The larger the crystallite, the narrower the melting range. In the case of sample PP-25G-C-E, the crystallite according to the XRD results is larger and the sample melts at 160.8 °C, but in a larger melting range (the difference between Tm and onset is 8.5 °C). In the case of sample PP-25G-E, the crystallite is smaller according to the XRD results and the sample melts at 161.5 °C, but in a narrow melting range (the difference between Tm and onset is 4.3 °C). The ΔHm/ΔHc are reduced significantly, as seen in Table 4, with the introduction of elastomer and slightly less with the introduction of treated ash. By adding treated ash, the degree of crystallinity does not change, and melting/crystallization enthalpy decreases by almost 7% compared to PP-25G. The addition of elastomer leads to an increase in the degree of crystallinity by almost 20% and a decrease in melting/crystallization enthalpy by almost 12% for PP-25G-E and by 20–27% for PP-25G-C-E. The degree of crystallinity of PP is calculated by the following equation:

$$X_{C}(\%)=\frac{∆H_{m}}{(1-\emptyset )∆H_{m}^{o}}\times 100\%$$

where $X_{C}$ is the crystallinity, $∆H_{m}$ is the apparent enthalpy of crystallization for the composite, $∆H_{m}^{o}$ is the enthalpy corresponding to the melting of 100% crystalline isotactic PP and ∅ is the weight fraction of the components incorporated in the polymer matrix; $∆H_{m}^{o}$ of PP is 209 J/g according to previous studies [42,43].

The degree of crystallinity, $X_{C}$, varies in the order PP-25G-C-E > PP-25G-E > PP-25G-C > PP30G > PP-25G and is influenced by the level of interaction among the components. These results are consistent with the results obtained by FTIR analysis for the crystallinity index. It is notable that the addition of elastomer leads to an increase in crystallinity, which, according to studies by Tjong et al. [44] and by Lin et al. [45], implies that SEBS accelerates the crystallization process by acting as a nucleating agent for PP and augments its crystallization. A study by Setz et al. [46] explains that strong interfacial bonding exists between PP and SEBS due to the chemical structure of PP being close to that of the midblock of SEBS. Usually, the degree of crystallinity determined by DSC should have been correlated with the degree of crystallinity determined by XRD. However, in our case, they do not correlate because the XRD analysis was performed on the injected samples at room temperature, the diffraction information being obtained for approximately 100 μm below the surface of the sample. As shown in Section 3.1, during the injection process complex, morphological structures are obtained depending on the influence of each component on the flow of the melt in the mold and the formation of crystallites (crystallization rate, crystallite orientation and size). In the case of DSC, we can no longer speak of the existence of complex morphological structures of extended crystals with a strong orientation in the flow direction (skin) or folded crystals, mainly non-oriented (core), as in the case of injection molding, which involves the flow of the polymer melt in a mold under certain conditions of temperature and pressure. For DSC analysis, the sample is subjected to a heating–cooling–heating cycle, after which the so-called “sample randomization” takes place. We can speak of a uniform spherulitic morphology with alpha spherulites of different size or disorder [28].

### 3.5. Mechanical and Dynamic Mechanical Analysis

**Figure 5:**
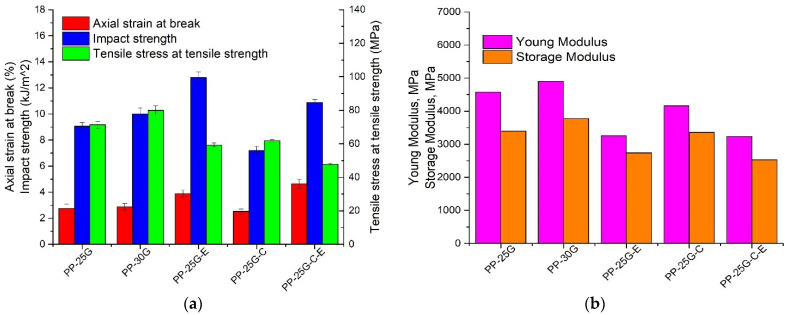

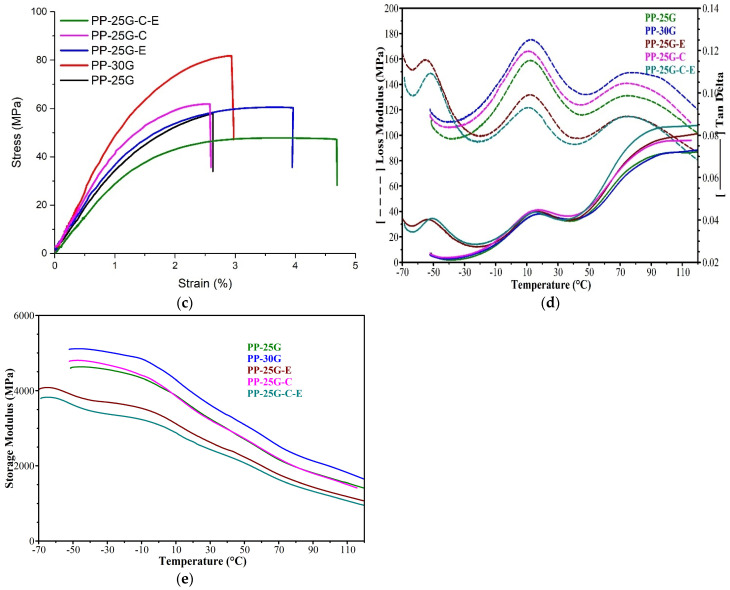
Mechanical and dynamic mechanical properties of PP-25G, PP-30G, PP-25G-E, PP-25G-and PP-25G-C-E composites. (**a**) Tensile and impact properties; (**b**) Young’s moduli and storage moduli at 25 °C; (**c**) stress vs. strain curves; (**d**) loss moduli and loss factors; (**e**) storage moduli.

Glass fiber-reinforced thermoplastic composites offer advantages such as high mechanical strength, hardness and unlimited shelf life together with a positive environmental impact, as shown in a study by Colucci et al. [47]. The variation in mechanical properties can mainly depend on the fiber shortening and breaking that takes place during the extrusion and injection molding processes. It is well known that glass fiber greatly increases the strength and stiffness of neat thermoplastic polymers [48,49,50]. In Figure 5a and Table 5, composite PP-25G-C-E, which contains glass fiber and elastomer as well as fly ash, is competing in its mechanical properties with both PP-25G and PP-30G. A key aspect that determines whether a material is suitable or not for an application in the automotive industry is its balance of mechanical properties, because by greatly improving one property, we risk downgrading another one. Therefore, for many automotive applications, the important properties for reinforced thermoplastic composites are improved toughness (impact strength and axial strain) in addition to high strength and stiffness. By looking at the variation in properties in Figure 5a,b as well as in Table 5, we see that while the introduction of elastomer leads to 25–39% higher impact strength, the values for stiffness (Young’s modulus and storage modulus E’) are reduced by approximately 30%. With the addition of 5% ash, the composite PP-25G-C-E has higher impact strength value than PP-25G of approximately 17%, than PP-30G of approximately 8%, as well as higher axial strain value of approximately 41% and 38%, respectively, as seen in Figure 5c. A particular case can be observed in the stress vs. strain curves that indicates a lower tensile strength for PP-25G-C-E compared to PP-25G-E while having higher axial strain. Sanporean et al. [23] reported in their study that the presence of elastomer may improve a composite’s toughness (expressed by axial strain and impact strength) at the cost of tensile strength. Their study also showed that various ratios between a PP matrix, a layered silicate and elastomer may lead to variations in mechanical properties where a decrease in tensile strength and increase in axial strain are observed. Their argument is that good particle dispersion and interactions between the components may lead to percolated structures that improved the mechanical properties of neat PP. It is possible that a similar effect might have occurred in our study. These results prove good dispersion and interaction among the components in PP-25G-C-E. A study by Tjong et al. [44] showed similar results and explained that the maleic anhydride functional group improves the interfacial adhesion between SEBS and glass fiber, thus improving its impact strength.

Through DMA analysis, the impact of ash powder and elastomer on the viscoelastic behavior of glass fiber-reinforced PP was analyzed. This allowed for an evaluation of the stiffness and elastic behavior via the storage modulus (E′) and the viscous characteristic through the loss modulus (E″) for the studied composites. Furthermore, the loss factor (tan δ) was obtained for assessing the impact resistance of the composites as well as the weight of the elastic and viscous phases [51]. Figure 5b displays the comparison of the storage modulus at 30 °C to Young’s modulus, while Figure 5d reveals the loss modulus and loss factor of the PP composites as a function of temperature. E′ of PP-25G-C-E decreases by 25–33% compared to PP-25G and PP-30G, respectively, and these values are consistent with their Young’s modulus values (Figure 5b). The loss factor and loss modulus curves of each composite are comparable. To distinguish the samples, two relaxations can be seen in the loss factor vs. temperature and loss modulus vs. temperature curves of PP composites reinforced with glass fiber or a mixture of glass fiber and treated fly ash. Another relaxation can be seen in Figure 5d exclusively on the curves for composites containing elastomer, PP-25G-E and PP-25G-C-E. E″ peak 1 is only found in PP-25G-E and PP-25G-C-E because it corresponds to the Tg of the elastomer (olefin block) [52], whereas E″ peak 2 is associated with the glass transition of PP (Tg) and E″ peak 3 is associated with the lamellar slip and rotation in the crystalline phase of PP [53]. The tan δ curves of the samples exhibit three distinct relaxation peaks that correspond to the peaks of the loss modulus. The loss modulus curve’s highest peak, which corresponds to Tg, is seen for PP-30G at approximately 13.4 °C, whereas the peak for PP-25G was found at approximately 11 °C. Table 6 indicates that PP-25G-E and PP-25G-C-E exhibited Tg values that were 0.1–1.7 °C higher than PP-25G. This suggests that there was an improvement in the viscous properties due to good component interaction. The PP glass transition temperature (Tg) of 17 °C was determined from the tan δ vs. temperature plot, while a second peak at around 95 °C is associated with lamellar slip and rotation in the crystalline phase of PP. Glass transitions of elastomer can be seen in Figure 5 (d) at −52.6 °C and −50.7 °C only for PP-25-G-E and PP-25G-C-E. Composites with elastomer have the lowest values for Tg (from tan delta) and the highest tan delta peak heights. These results mean lower thermal stability for these samples, but higher impact strength. The DMA results correlate very well with mechanical impact tests (Figure 5a) and with the TGA results (Table 3). Figure 5e shows the storage moduli of the studied composites as a function of temperature. Sample PP-30G has the highest stiffness of all samples due to having the highest concentration of incorporated glass fiber in neat PP [54]. PP-25G-C has higher stiffness compared to PP-25G due to the presence of well dispersed ash powder and compatibilizing agent in the PP system. Such an enhancement was pointed out in a study by Zoukrami et al. [41], where more effectively dispersed silica particles and the presence of PP-MA compatibilizing agent caused stronger interactions and increases in the elastic moduli of the materials. A similar effect with the introduction of well-dispersed ash powder and compatibilizing agent into recycled PP was observed in a previous study by Teodorescu et al. [12].

### 3.6. Nanomechanical Analysis

**Figure 6:**
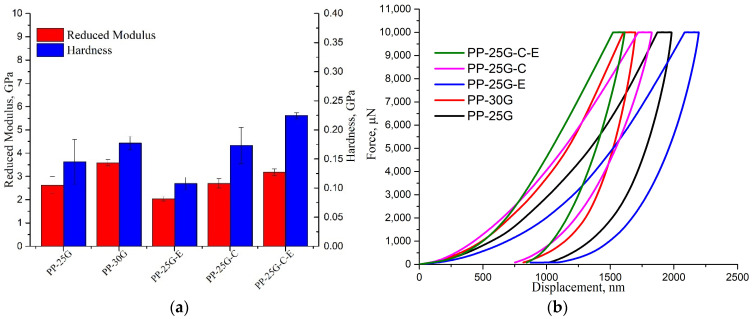
Nanomechanical properties of PP-25G, PP-30G, PP-25G-E, PP-25G-C and PP-25G-C-E composites; (**a**) reduced moduli and hardness; (**b**) force vs. displacement curves.

Increase in reduced modulus and hardness can be seen in Figure 6a by approximately 27% and 18%, respectively, for PP-30G compared to PP-25G. This increase is caused by the presence of 5% extra glass fiber. When adding elastomer, we see a decrease for PP-25G-E of approximately 22% for modulus and 25% for hardness compared to PP-25G caused by an increase in surface elasticity. This behavior is confirmed by the force–displacement curves in Figure 6b, where PP-25G-E has the highest elastic recovery out of all the composites. The presence of fly ash in PP-25G-C leads to an increase in hardness of 16% for this composite compared to PP-25G. In a previous article written by Teodorescu et al. [12], similar behavior was observed where the introduction of silico-aluminous industrial waste in recycled polypropylene led to an increase in reduced moduli and hardness for the composites with ash powder compared to neat PP. Other studies by Enrique et al. [55], Shokrieh et al. [56] and Alateyah et al. [57] also showed that the addition of fillers such as exfoliated graphite nanoplatelets or nano-magnesia led to an increase in reduced moduli and hardness compared to neat polymer, while a study by Aldousiri et al. [58] demonstrated increases in hardness and reduced moduli for the studied composites with the introduction of 5 wt.% layered silicate. Composite PP-25G-C-E showed the highest increase in nanomechanical properties with 18% for reduced modulus, 35% for hardness compared to PP-25G and 21% increase in hardness compared to PP-30G. These results indicate that PP-25G-C-E has high surface resistance and would be suited for exterior applications in industries such as the automotive industry.

The measures of Young’s modulus and reduced modulus, while related, are distinctive mechanical properties determined via different methods. Tensile or compressive tests are utilized to find Young’s modulus, whereas the reduced modulus is acquired through indentation or nanoindentation processes. Both metrics offer an understanding of the stiffness of a material, but discrepancies may occur among samples because of the differences in testing methods, load conditions, sample preparation, and inherent material characteristics. Even though there might be some connection between these two moduli, it is not certain that their variation will be similar across different samples. A complex study by Nardi et al. [59] showed how important parameters such as depth and morphology are when determining the hardness and reduced modulus of a nanoparticle-reinforced nanocomposite. Through their work, it was revealed that high surface concentrations of nanoparticles do not necessarily lead to stiff nanocomposites. This would explain why the presence of C in PP-25G-C led to an increase in reduced modulus but not in storage or Young’s modulus when compared to PP-25G. The XRD results showed that the ash particles oriented on the surface in a parallel plane led to increased crystallinity and orientation of the alpha PP crystallites. This behavior justifies the improvement in the surface resistance to the deformation produced by the penetration of the Berkovich probe at a depth of less than 2500 nm. The elastomer has the opposite effect compared to the ash (it decreases the crystallinity and orientation of the alpha PP crystallites), which is reflected in a decrease in the surface properties of PP-25G-E. In the case of PP-25G-C-E, the strong interaction between C, E, PP-MA, and PP led to the orientation of ash particles on the surface in a parallel plane, while the elastomer particles were distributed either as transcrystalline layers on the surface or in the form of radial spherulites inside the sample. This behavior confirms the improvement in surface properties.

### 3.7. SEM and EDX Analysis

**Figure 7:**
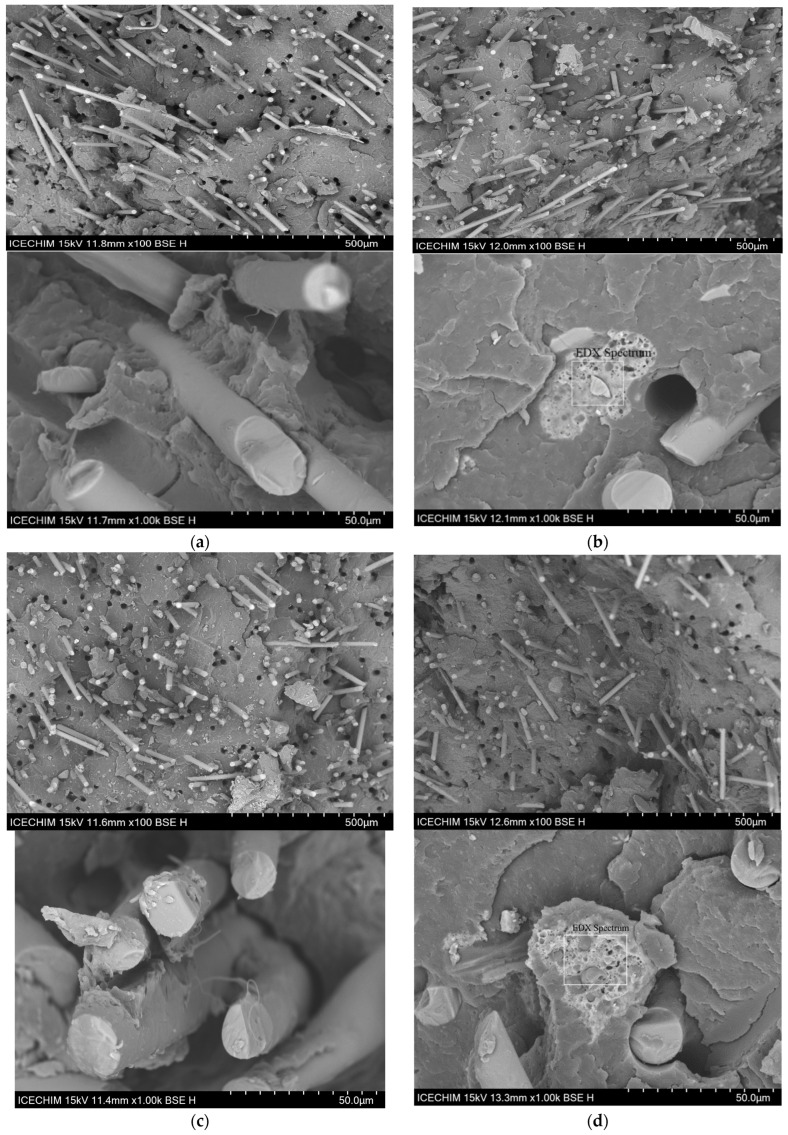
SEM surface morphology (100×) and (1000×) of (**a**) PP-25G; (**b**) PP-25G-C composite; (**c**) PP-25G-E composite; (**d**) PP-25G-C-E composite.

Figure 7a shows the morphology of PP-25G, where the glass fiber can be clearly seen embedded in the polymer matrix. It is noticeable throughout every sample that holes can be seen uniformly distributed, which would suggest that the glass fibers are well dispersed in the polymer matrix. Material from the polymer matrix can be seen on the surface of the glass fibers after the fracture in Figure 7c ×1000, evidence of good adhesion between the fiber and the polymer matrix. Studies by Fu et al. [60] and Thomason et al. [61] found that the amount of residual polymer still attached to the fibers can indicate the state of fiber–matrix adhesion. The addition of elastomer seems to prevent the pullout of the glass fibers (more organic material present on the fibers), the glass fibers being better anchored in the polymer matrix, as seen in Figure 7c, which correlates with previous analysis from this study that showed a strong interaction between PP, maleic anhydride and SEBS [45]. It can also be pointed out that the shape of the holes left by pulling out the glass fibers do not have perfectly round shapes. This indicates that the organic material from the matrix remains attached to the fibers when pulled out. With the addition of ash powder, agglomerates with dimensions of 45–50 µm can be seen in Figure 7b,d. However, it should be noted that the ash particles are well embedded in the polymer matrix, as no separation can be seen and the pullout effect is decreased compared to PP-25G (percentage of holes reduced and the irregular shape of the holes). EDX analysis (Table 7) confirms that the agglomerates are made of ash powder particles, as their elemental composition coincides with the chemical oxidic composition of the ash powder [12].

## 4. Conclusions

Through melt processing under dynamic conditions, composites based on PP reinforced with long glass fibers (G), thermal power plant ash and thermoplastic elastomer (linear triblock copolymer) (E) were obtained. The effect of the components, both individually and together, on the morphological, thermal, and (nano)mechanical properties of polypropylene reinforced with long glass fibers was analyzed. Each analysis was a necessary step in showcasing the degree of interactions between the components in the studied composites. Firstly, morphostructural analysis showed strong interactions between the components in each composite and confirmed their presence in the polymer matrix. Thermal analysis indicated that the presence of E accelerated the crystallization process by acting as a nucleating agent for PP. From a mechanical point of view, by mixing 20 wt.% E with 25 wt.% G-reinforced PP (PP-25G), an increase in toughness (increase in impact strength and axial strain by 39% and 18%, respectively) was obtained, but at the expense of stiffness, which decreased by approximately 25%. The addition of 5 wt.% treated ash (C) in PP-25G had very little influence on the stiffness (it decreased by less than 10%). On the other hand, the impact strength and tensile strength decreased by 20–30%. The combined effect of toughening with E and stiffening with C was achieved in the PP–G–E–C composite. PP-25G-C-E presented higher impact strength and axial strain compared to PP-25G and even PP-30G, proving good dispersion and interaction among components. Nanomechanical properties of PP-25G-C-E also showed an increase in surface hardness (21–35%) compared to PP-25G and PP-30G, indicating high surface resistance that would be suited for exterior applications in industries such as the automotive industry. Thus, a low-cost yet efficient alternative was developed using polypropylene reinforced with long glass fibers, elastomer and fly ash, with potential for use in industry. The process involved the replacement of a quantity of glass fibers with surface-treated ash and the addition of thermoplastic elastomer, without adversely affecting the specific properties required for the use of such polymer composites in the automotive industry. The process resulted in an enhancement in both toughness and surface properties, making the PP-25G-C-E composite a viable alternative to the regularly used glass fiber-reinforced polypropylene. Due to its increased toughness, composite PP-25G-C-E may find applications in the automotive industry, specifically in car bumpers, which are required to absorb impact energy and have higher resistance to crack initiation.

## Figures and Tables

**Table 1:** The PP composite formulations.

Sample	PP (wt.%)	MA (wt.%)	C (wt.%)	E (wt.%)	G (wt.%)
PP-25G	75		-	-	25
PP-30G	70		-	-	30
PP-25G-E	52.5	2.5	-	20	25
PP-25G-C	66	2.5	6.5	-	25
PP-25G-C-E	46	2.5	6.5	20	25

**Table 2:** XRD results for the studied PP composites.

		αPP (110)	αPP (040)	αPP (130)	αPP (111)	αPP (13-1)	SiO_2_ (101)	IO
PP-25G CI = 66%	2θ	14.096	16.942	18.597	21.212	21.889		0.911
	d (Å)	6.277	5.229	4.767	4.185	4.057		
	Height (cps)	16,666	15,184	7433	6909	6201		
	Size (Å)	120	152	111	78	119		
	FWHM (°)	0.663	0.5273	0.7244	1.0299	0.6748		
PP-25G-C CI = 70%	2θ	14.096	16.927	18.591	21.127	21.819	26.55	1.368
	d (Å)	6.277	5.233	4.768	4.201	4.069	3.3539	
	Height (cps)	16,998	23,259	8362	3268	7568	690.12	
	Size (Å)	128	149	110	124	94	215.31	
	FWHM (°)	0.6252	0.5363	0.7297	0.6506	0.8597	0.4031	
PP-25G-E CI = 63%	2θ	14.081	16.911	18.576	21.136	21.876		0.964
	d (Å)	6.284	5.238	4.772	4.2	4.059		
	Height (cps)	16,645	16,044	8270	5958	6315		
	Size (Å)	122	148	107	74	110		
	FWHM (°)	0.6546	0.5426	0.749	1.0852	0.732		
PP-25G-C-E CI = 58%	2θ	14.07	16.896	18.517	21.131	21.828	26.5355	1.025
	d (Å)	6.286	5.243	4.787	4.200	4.068	3.356	
	Height (cps)	11,862	12,157	6002	4175	7052	669.3	
	Size (Å)	121	146	109	115	94	225.69	
	FWHM (°)	0.6572	0.5466	0.733	0.7003	0.8559	0.3616	

**Table 3:** TGA results of E, PP-25G, PP-30G, PP-25G-E, PP-25G-C and PP-25G-C-E composites.

Sample	RT—230 °C Wt. Loss %	Onset Point Temp °C	Tmax °C	Residue 700 °C	Temp for Wt. Loss 5% °C
E	0.03	418.8	445.2	0.15	407.2
PP-25G	0.27	430.8	457	25.32	401.4
PP-30G	0.28	430.1	455.7	29.46	403.6
PP-25G-E	0.16	397.9	433.2	23.06	371.9
PP-25G-C	0.36	430.5	456.7	27.34	396.9
PP-25G-C-E	0.36	416.5	440.9	23.32	395.7

**Table 4:** DSC results for PP-25G, PP-30G, PP-25G-E, PP-25G-C and PP-25G-C-E composites.

Sample	Cooling, Crystallization				Melting, 2nd Heating			
	Onset	Tc	ΔHc	Xc	Onset	Tm	ΔHm	Xc
	°C	°C	J/g	%	°C	°C	J/g	%
PP-25G	124.8	122.4	78.4	50.01	156.8	161.5	69.55	44.37
PP-30G	124.9	122.3	74.2	50.71	156.8	161.1	65.57	44.81
PP-25G-E	124.8	122.2	69	60.02	157.2	161.5	61.09	53.14
PP-25-G-C	124.9	122.1	72.5	49.55	156.8	161.5	64.85	44.32
PP-25G-C-E	127.2	122.6	57.07	54.61	152.3	160.8	55.63	53.23

**Table 5:** Mechanical properties of PP-25G, PP-30G, PP-25G-E, PP-25G-C and PP-25G-C-E composites.

Sample	Tensile Stress at Tensile Strength (MPa)	Young’s Modulus (MPa)	Axial Strain at Break (%)	Impact Strength (KJ/m^2^)
PP-25G	71 ± 2	4576 ± 575	2.8 ± 0.3	9 ± 0.3
PP-30G	80 ± 3	4905 ± 435	2.9 ± 0.3	10 ± 0.5
PP-25G-C	62 ± 0.5	4162 ± 199	2.5 ± 0.2	7 ± 0.3
PP-25G-E	59 ± 1.2	3258 ± 262	3.9 ± 0.3	13 ± 0.5
PP-25G-C-E	48 ± 0.5	3238 ± 115	4.6 ± 0.3	11 ± 0.2

**Table 6:** Storage modulus, loss modulus and loss factor values for PP composites.

Sample	Storage Modulus, E′		Loss Modulus, E″						Loss Factor					
	Temp	E′	Temp	E″ Peak1	Temp	E″ Peak2	Temp	E″ Peak3	Temp	Tan Delta	Temp	Tan Delta	Temp	Tan Delta
	°C	MPa	°C	MPa	°C	MPa	°C	MPa	°C	Tan δ Peak 1	°C	Tan δ Peak 2	°C	Tan δ Peak 3
PP-25G	25	3393	-	-	11.5	159	74.6	131.3	-	-	17.2	0.043	100.9	0.072
PP-30G	25	3775	-	-	13.4	175.4	78.1	149.3	-	-	18.4	0.043	98.0	0.071
PP-25G-C	25	3358	-	-	10.5	166.4	74.6	140.9	-	-	16.7	0.045	99.8	0.077
PP-25G-E	25	2739	−53.5	159.1	13.2	131.9	74.1	114.9	−52.6	0.040	17.6	0.044	93.6	0.078
PP-25G-C-E	25	2528	−52.3	148.9	11.6	121.8	75.4	114.7	−50.7	0.041	15.6	0.043	92.6	0.083

**Table 7:** EDX analysis data.

Sample	Element	%Weight	Standard Deviation, σ
PP-25G-C	C	22	1
	Si	15.8	0.3
	Au	10.5	0.4
	Al	7.4	0.2
	Fe	3	0.2
	Ca	2.1	0.1
	K	1.5	0.1
	Mg	0.9	0.1
	Na	0.2	0.1
PP-25G-C-E	C	20.9	1.5
	Si	16.5	0.4
	Au	9	0.5
	Al	7.8	0.2
	Fe	5.9	0.4
	Ca	1.4	0.1
	K	1.3	0.1
	Mg	0.8	0.1

## Data Availability

Data are contained within the article.
